# Andrographolide and Its Derivatives: A Comprehensive Review of Anti-Infective Properties and Clinical Potential

**DOI:** 10.3390/molecules30214273

**Published:** 2025-11-03

**Authors:** Zimo Ren, Zihan Chen, Yuhan Xie, Paolo Coghi

**Affiliations:** 1 School of Pharmacy, Macau University of Science and Technology, Macau 999078, China; 2230028575@student.must.edu.mo (Z.R.); zhchen@must.edu.mo (Z.C.); 2240026307@student.must.edu.mo (Y.X.); 2 State Key Laboratory of Mechanism and Quality of Traditional Chinese Medicine, Macau University of Science and Technology, Macao 999078, China

**Keywords:** andrographolide, anti-infective, andrographolide derivatives, antiviral, antibacterial, antiparasitic

## Abstract

*Andrographis paniculata*, a medicinal plant widely found in Asia, contains andrographolide as its main active compound, known for its wide-ranging pharmacological effects, including anti-inflammatory, anti-cancer, anti-obesity, and anti-diabetic properties. Recent investigations have highlighted the anti-infective potential of andrographolide and its derivatives, with demonstrated antiviral, antibacterial, and antimalarial activities. This review summarizes progress in andrographolide’s anti-infective applications, focusing on its structure–activity relationship (SAR) and mechanisms of action. Researchers have used semi-synthetic methods, such as esterification, oxidation, Michael addition, salification, and hybrid design, to enhance andrographolide’s physicochemical properties and biological activity. These derivatives show potent antiviral activity against RNA and DNA viruses, antibacterial activity against Gram-positive and Gram-negative bacteria, antifungal effects, and antiparasitic activity against *Plasmodium* spp. and *Leishmania* spp. Nevertheless, poor solubility and limited bioavailability still hinder their clinical translation. Strategies such as nano delivery systems and β-cyclodextrin complexes are discussed to improve bioavailability. Although andrographolide itself has not received regulatory approval as a stand-alone drug, several andrographolide-containing preparations have been clinically used in certain countries. Overall, this review brings together evidence on antiviral, antibacterial, antifungal, and antiparasitic activities, linking them with structure–activity trends and pharmacokinetic insights, thereby providing a consolidated foundation for future development and clinical translation.

## 1. Introduction

*Andrographis paniculata* [1], a medicinal plant widely distributed across Asia, has been traditionally employed for the treatment of inflammatory disorders [2] and infectious diseases [3]. Its main active ingredient, andrographolide, the principal bioactive constituent, exhibits anti-inflammatory activity [4], battling cancer [5,6,7], curbing obesity [8,9], and managing diabetes [10,11]. In anti-infective contexts, andrographolide and selected derivatives modulate pathogen-induced inflammatory signaling (e.g., NF-κB/TLR pathways) and, in several models, directly inhibit pathogen replication [12,13,14].

The major bioactive constituent, andrographolide, is isolated primarily from the leaves and stems and features a bicyclic diterpene scaffold with an α,β-unsaturated γ-lactone and conjugated double bonds—structural motifs associated with its biological activities [15,16]. Recent research highlights how it combats infections by boosting the immune system [17], dialing down inflammation [18], and clearing out oxidative stress [19]. Considerable progress has also been made in elucidating its antiviral, antibacterial, and antimalarial mechanisms of action [20].

We provide an integrated appraisal of the anti-infective properties of andrographolide and its derivatives across viruses, bacteria, fungi, and parasites, explicitly connecting mechanistic insights and SAR with formulation strategies and PK liabilities—a scope that, to our knowledge, has not been comprehensively consolidated. Inclusion criteria were original research articles reporting in vitro, in vivo, or clinical outcomes for andrographolide or chemically defined derivatives within the last ~20 years. Exclusion criteria comprised narrative reviews, studies unrelated to infectious indications, derivatives not based on the andrographolide scaffold, duplicate records, and reports lacking primary quantitative endpoints (e.g., EC_50_/IC_50_, MIC, CC_50_, SI).

Literature was identified from the Web of Science Core Collection (cross-checked with PubMed/Scopus) using keywords related to andrographolide, derivatives, and infectious indications over 2005–2025. Screening followed the inclusion/exclusion criteria stated above. Data were organized by pathogen class with consistent reporting of EC_50_/IC_50_ (μM), CC_50_ (μM), MIC (μg·mL^−1^), and SI (CC_50_/EC_50_ or IC_50_).

In anti-infective research, andrographolide and its derivatives play important roles by inhibiting pathogen-induced inflammation, modulating the immune system, and scavenging oxidative stress. For example, andrographolide can inhibit inflammation caused by bacterial infection by regulating the mitogen-activated protein kinase (MAPK) and nuclear factor-κB (NF-κB) signaling pathways [21], thereby reducing the expression of pro-inflammatory cytokines. In antiviral activity, andrographolide can inhibit viral replication and regulate the expression of Nrf2 and its downstream genes [22], reducing lung inflammation caused by viral infection. In antimalarial activity, andrographolide, in combination with curcumin, has shown significant antiplasmodial activity against *Plasmodium falciparum* [23,24,25].

**Figure 1:**
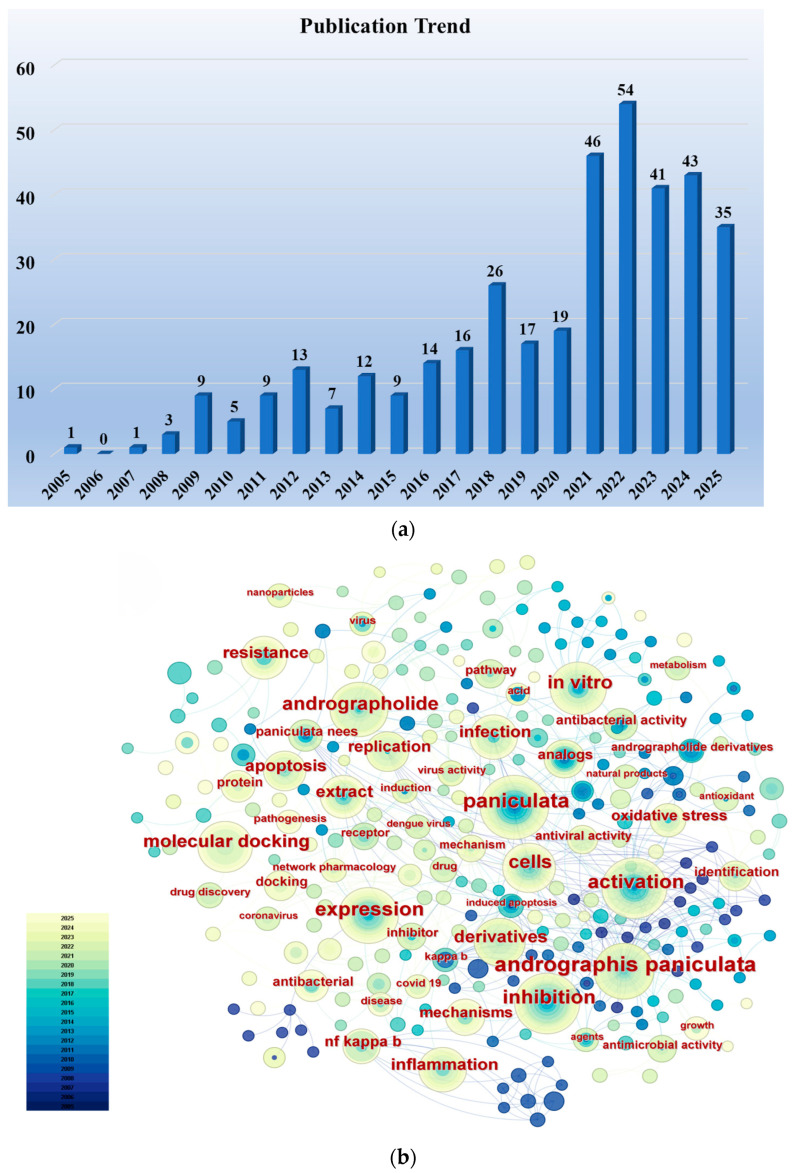
(**a**) Publication trends in research articles concerning the anti-infective activity of andrographolide over the past two decades. (**b**) Frequency distribution of keywords in research articles on the anti-infective activity of andrographolide over the past two decades. Supported by CiteSpace 6.4.R1.

We conducted a literature search in the Web of Science Core Collection using the keywords “Andrographolide” and “Anti-infective (including antiviral, antibacterial, antiparasitic)” (considering only research articles from the past two decades), identifying a total of 380 relevant research papers. Figure 1a illustrates the publication trend over the past two decades for research papers concerning the anti-infective activity of andrographolide. Although the number of papers published in certain years showed a slight decrease compared to the preceding year, the overall trend is upward, reflecting researchers’ increasingly intense interest in the anti-infective activity of andrographolide. Using CiteSpace software, an analysis of keyword frequency was conducted on these publications. Figure 1b clearly shows that keywords such as “infection”, “antibacterial activity”, “antiviral activity”, “derivatives”, “extract”, and “molecular docking” appear with high frequency. This indicates that current researchers’ interests are primarily focused on the biological activity of andrographolide, the synthesis of its derivatives, and structure–activity relationships.

The scope of this review covers viruses, bacteria, fungi, and parasites, and the reporting standards for potency and selectivity are summarized in Table 1.

## 2. Structural Diversity and Representative Synthetic Derivatives

### 2.1. Parent Skeleton and Key Functional Groups

**Figure 2:**
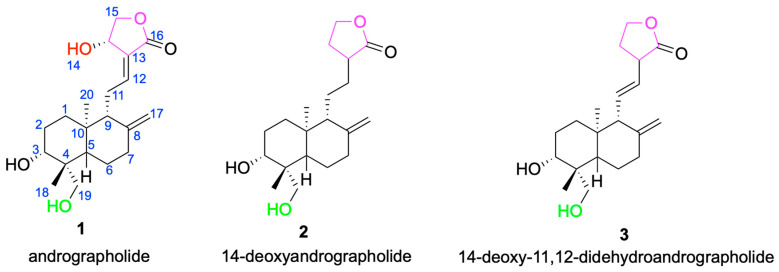
Chemical structures of andrographolide **1** and its analogs **2** and **3**.

The core framework of andrographolide is a rigid, lipophilic C20-labdane-γ-lactone scaffold, in which a decahydronaphthalene ring system is fused to a γ-butyrolactone moiety through a C-8/C-17 conjugated double bond [33]. This architecture influences molecular conformation and membrane permeability and provides a stable stereochemical platform for downstream derivatization [34]. In addition to the parent scaffold andrographolide **1**, other compounds such as 14-deoxyandrographolide **2** and 14-deoxy-11,12-didehydroandrographolide **3** have also been isolated from *Andrographis paniculata* [35] (Figure 2). These compounds share structural similarities with andrographolide but exhibit differences at certain positions, which may influence their biological activities. For example, 14-deoxyandrographolide 2 lacks the hydroxyl group at C-14, while 14-deoxy-11,12-didehydroandrographolide 3 has a double bond at the C-11 and C-12 positions. These structural features may endow them with unique antiviral and antibacterial activities [36,37].

The primary hydroxyl group at C-14 is readily esterified or oxidized to form α, β- unsaturated diketone or sulfonate esters, which can form covalent bonds with viral proteases such as the cysteine residue of SARS-CoV-2 [38]. The primary hydroxyl group at C-19 can be converted into sodium sulfonate or phosphate salts [39]. For example, the sulfonated preparation Xiyanping injection (sodium andrographolide sulfonate) is used clinically in China for acute upper respiratory tract infections (e.g., acute pharyngotonsillitis) and acute bronchitis. This herbal-derived product is not equivalent to regulatory approval of pure andrographolide, and the supporting clinical evidence remains limited in scope [40].

### 2.2. Overview of Semi-Synthetic Strategies: Esterification, Oxidation, Michael Addition, Salification, and Hybrid Design

#### 2.2.1. Esterification

Esterification at C-14/C-19 can increase aqueous solubility and tune lipophilicity; C-14 ester chain length correlates with influenza A antiviral potency in multiple series. This modification markedly improves the aqueous solubility of andrographolide and enhances its bioavailability [41,42]. For instance, compound 12 (AL-1), a C-14/C-19–modified conjugate, markedly reduced the viral burden of influenza A (H1N1) (EC_50_ = 7.2 μM; SI = 109) in vitro and protected mice at high dose (see Section 3.1, H1N1).

#### 2.2.2. Oxidation

Oxidations that install α,β-unsaturated carbonyls can enhance cysteine-targeting electrophilicity, with selectivity and safety trade-offs. This strategy can introduce a variety of active functional groups, thereby modulating the pharmacological properties and pharmacokinetic characteristics of the drug [43,44]. For example, the α, β-unsaturated carbonyl derivative 14-deoxy-11,12-didehydroandrographolide exhibited enhanced cysteine-targeting reactivity in biochemical assays, illustrating the activity–selectivity trade-off (see Section 2.1 and Section 3.1).

#### 2.2.3. Michael Addition

Michael acceptor-containing analogues may improve enzyme inhibition but require reactivity control to mitigate off-target liabilities. This strategy can significantly enhance the biological activity of andrographolide, especially its antibacterial and antiviral activities [45,46]. For example, compound **10** (Andro-NBD) inhibits SARS-CoV-2 Mpro (IC_50_ = 2.79 ± 0.30 μM), whereas the non-Michael Compound **11** (NCTU-048) shows no measurable inhibition (IC_50_ > 240 μM), underscoring the need to control electrophilic reactivity (Section 3.1, SARS-CoV-2).

#### 2.2.4. Salification

Salification forms salts to improve aqueous solubility and sometimes solid-state stability. However, improved solubility does not guarantee better pharmacokinetics (e.g., oral bioavailability or half-life), which remain route- and formulation-dependent and require empirical confirmation [47]. For example, the sulfonated preparation Xiyanping improves aqueous solubility and is used clinically in certain regions as a herbal-derived product; however, this does not constitute regulatory approval of pure andrographolide, and any PK improvement requires empirical confirmation (Section 2.1 and Section 4).

#### 2.2.5. Hybrid Design

Hybridization (e.g., quinoline– or oxindole–labdane) enables dual-mechanism profiles and broader spectra while retaining the andrographolide pharmacophore. This strategy can significantly enhance the biological activity of andrographolide and expand its range of applications [48]. For example, the quinolinoxy–labdane compounds **14**/**15** display anti-flavivirus activity (ZIKV EC_50_ = 4.5 μM and 1.3 μM; SI = 19.7 and 17.5), and the oxindole–labdane Compound **16** exhibits anti-CHIKV activity (see Section 3.1, DENV/ZIKV and CHIKV).

By employing these semi-synthetic strategies, the physicochemical properties and biological activities of andrographolide can be effectively improved, providing more possibilities for its application in anti-infective research. Collectively, these strategies enrich the structural diversity of andrographolide derivatives and provide a rational framework for their clinical application.

## 3. Pathogen-Classified Anti-Infective Profile and Mechanisms

### 3.1. Antiviral Activity

Pathogen coverage and reporting standards: This section organizes findings by pathogen class, including viruses (SARS-CoV-2, influenza A/H1N1, DENV, ZIKV, CHIKV, EV-A71, HBV, HSV-1/2), bacteria (*Staphylococcus aureus* [MRSA], *Enterococcus faecalis*, *Escherichia coli*, *Pseudomonas aeruginosa*), and representative fungi (*Candida albicans*, *Candida auris*, *Aspergillus fumigatus*) and parasites (*Plasmodium falciparum*, *Leishmania donovani*). Potency is reported as EC_50_/IC_50_ (μM) or MIC (μg·mL^−1^) with corresponding CC_50_ (μM) when available. Selectivity index (SI) is defined as CC_50_/EC_50_ (or IC_50_), unless otherwise stated. To facilitate cross-study comparisons, we note key assay parameters (cell line, MOI, exposure time) where provided.

#### 3.1.1. RNA Viruses

##### Anti-SARSCoV2 Activity

SARS-CoV-2, first recognized in late 2019, is a betacoronavirus that caused the COVID-19 pandemic. A related bat coronavirus (RaTG13) shares ~96% genome sequence identity, and SARS-CoV-2 uses the angiotensin-converting enzyme 2 (ACE2) as the entry receptor. The virus uses the angiotensin-converting enzyme 2 (ACE2) as its receptor to enter human cells. It has an incubation period of about 5 days, with symptoms like fever, dry cough, and fatigue. Severe cases may develop into acute respiratory distress syndrome (ARDS) and require intensive care. SARS-CoV-2 spreads primarily through respiratory droplets and close contact between hosts As of late September 2025, the WHO COVID-19 Dashboard reports approximately 7.7 × 10^8^ confirmed cases and 7.1 × 10^6^ deaths worldwide (World Health Organization, 2025) [49,50,51].

Andrographolide inhibits the SARS-CoV-2 main protease (Mpro) in enzymatic assays with low-micromolar IC_50_ values, and available probe/labeling studies are consistent with cysteine engagement at Cys145. However, biochemical potency alone does not establish in vivo antiviral efficacy [52].

**Figure 3:**
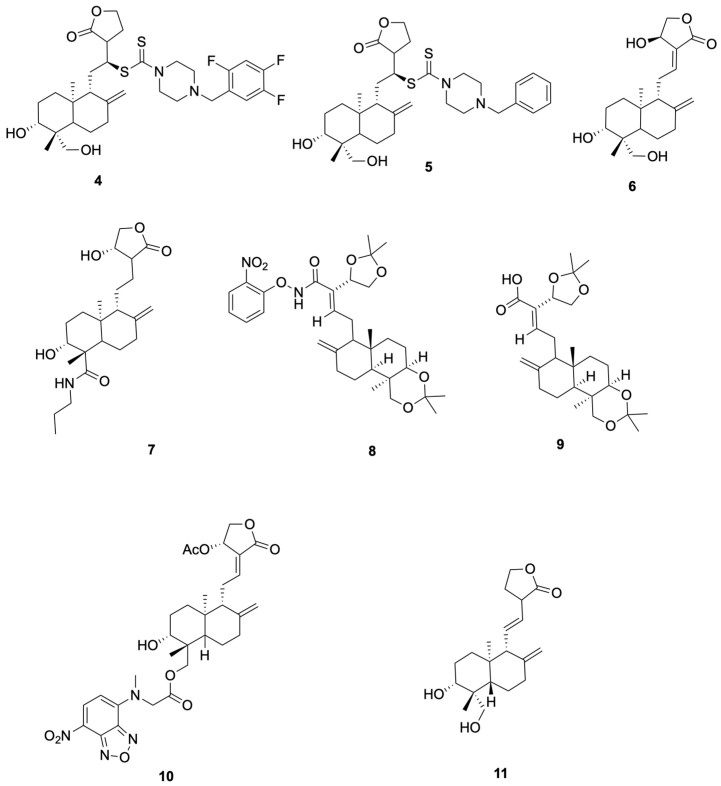
Compounds **4**–**11**.

Schulte et al. [53] synthesized compounds **4** and **5**, which showed anti-SARS-CoV-2 activity (representative antiviral andrographolide derivatives are shown in Figure 3). In Vero-E6 assays, Compound 4 reduced SARS-CoV-2 replication with NT_50_ = 8.1 μM and showed no detectable cytotoxicity at the tested concentrations (CC_50_ > 10 μM). It activates the KEAP1–NRF2 pathway with an ARE specificity ratio (SR) of 10.8. Compound **5** was an NRF2 activator (SR = 13.3) and showed no measurable antiviral effect up to 10 μM (NT_50_ > 10 μM), with CC_50_ > 10 μM. This derivative also exhibited improved solubility with low cytotoxicity, supporting further evaluation rather than establishing clinical candidacy.

Suriya et al. [54] synthesized and evaluated Compound **6** (14β-andrographolide) and Compound **7** showed cell-based antiviral activity against SARS-CoV-2 in Vero E6 plaque-reduction assays (NT50 = 2.1 μM and 3.7 μM, respectively), without cytotoxicity ≤ 10 μM (representative antiviral andrographolide derivatives are shown in Figure 3). Mechanistic data in that study associated the antiviral effect with KEAP1/NRF2 pathway modulation, rather than direct main protease inhibition. We therefore use **6** and **7** as representative examples in this section and describe their activity as host-directed antiviral effects in cell assays.

Rahman et al. [55] synthesized and profiled Compound **8** and Compound **9** as β-glucosidase inhibitors (representative antiviral andrographolide derivatives are shown in Figure 3). Compound **8** showed the best activity in the series (IC_50_ = 92.4 μM), outperforming castanospermine (108.6 μM), consistent with an electron-withdrawing *o*-NO_2_ effect on the aryl group. Compound **9** exhibited moderate inhibition (IC_50_ = 102.4 μM), comparable to the simple phenyl amide (106.5 μM) and better than andrographolide (142.5 μM). As these readouts derive from a biochemical model (sweet-almond β-glucosidase), enzyme-level potency is hypothesis-generating; translation to anti-infective efficacy requires cellular and in vivo confirmation.

**Figure 4:**
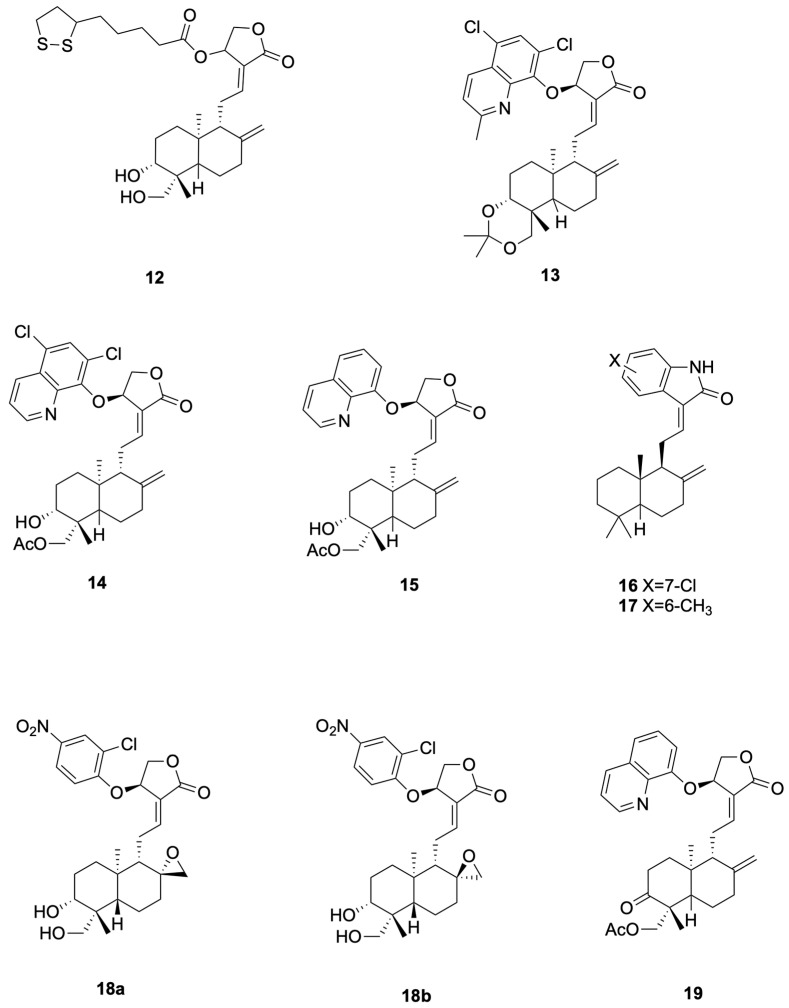
Compounds **12**–**19**.

Shi et al. [52] reported that compound **10** (Andro-NBD) inhibits SARS-CoV-2 Mpro in a biochemical assay (IC_50_ = 2.79 ± 0.30 μM) and labels the enzyme in gel; the signal is abolished by the C145A active-site mutant, and MS indicates an adduct on Cys145, consistent with a putative covalent mechanism. In contrast, the thiol-insensitive analogue compound **11** (NCTU-048) shows weak inhibition (>~240 μM), supporting cysteine dependence. As these are enzyme-level readouts, potency and labeling are hypothesis-generating; antiviral efficacy requires cellular and in vivo confirmation (see Figure 4 for representative antiviral derivatives).

##### Anti-Influenza A (H1N1) Virus Activity

Influenza A (H1N1), which caused the 2009 global pandemic, remains a major respiratory pathogen worldwide. It originated from pigs and is also known as “swine flu.” The virus is highly contagious and can cause respiratory infections with symptoms like fever, cough, and sore throat. The 2009 H1N1 pandemic was notable for its rapid spread and affected many countries. Vaccines and antiviral medications were used to control its impact. Since then, H1N1 has become a seasonal flu virus and continues to circulate globally [56].

In the case of influenza A virus, andrographolide derivatives have demonstrated the capacity to inhibit the RNA-dependent RNA polymerase (RdRp), which is a key enzyme for viral RNA synthesis. By targeting RdRp, these compounds can effectively halt the production of viral RNA, thus limiting the spread of the virus within the host. The C-14 ester chain length of andrographolide derivatives appears to correlate with antiviral potency against influenza A, with optimal chain lengths enhancing the interaction with the viral enzyme.

Chen et al. [57] synthesized compounds **12**, which demonstrated potent anti-H1N1 activity (Figure 4 presents representative antiviral andrographolide derivatives). **12** (CC_50_ = 784 μM, EC_50_ = 7.2 μM, SI = 109) completely prevented mortality in mice at 200 mg kg^−1^ day^−1^, reduced lung virus titers by 46%, and inhibited viral adsorption to erythrocytes by directly interfering with hemagglutinin–receptor interactions. **12** showed CC_50_ = 784 μM and SI = 109 (EC_50_ ≈ 7.2 μM in the same assay). The 200 mg·kg^−1^·day^−1^ dose used in mice represents a relatively high pharmacological exposure and warrants pharmacokinetic characterization to establish clinical relevance.

##### Anti-Dengue and Anti-Zika Viruses’ Activity

Dengue is a significant infectious disease affecting public health across tropical and subtropical areas worldwide. It presents clinically with a biphasic fever pattern, accompanied by symptoms such as severe headache, muscle and joint pain, profound fatigue, skin rash, swollen lymph nodes, and a marked reduction in white blood cell count [58,59].

Zika virus (ZIKV), a member of the Flavivirus genus within the Flaviviridae family, is classified as an arthropod-borne virus (arbovirus). Its primary mode of transmission to humans is through the bite of infected mosquitoes. Clinically, Zika fever often presents with nonspecific symptoms, making it challenging to distinguish from other arboviral diseases such as dengue and chikungunya, and frequently leading to potential misdiagnosis [60].

For dengue virus and zika virus, andrographolide derivatives have been found to interfere with the viral RdRp and Mpro, like their action against other RNA viruses. These interactions prevent the virus from efficiently replicating its RNA genome and processing its polyproteins. The α, β-unsaturated lactone moiety of andrographolide derivatives is particularly effective in covalently binding to viral targets, thereby inactivating them and reducing viral replication.

In this review, SI is defined as CC_50_/EC_50_ (or IC_50_), unless otherwise stated. Where EC_50_ values are ≥10–20 μM (e.g., DENV 22.6 μM; ZIKV 27.9 μM for the 14-aryloxy analogue), we describe the antiviral activity as weak-to-moderate rather than “potent”, and we report EC_50_, CC_50_, and SI (=CC_50_/EC_50_) explicitly to avoid ambiguity.

Zhou et al. [61] synthesized the 14-aryloxy analogue compounds **13** (Figure 4 presents representative antiviral andrographolide derivatives), which showed dual activity with weak-to-moderate potency against DENV (EC_50_ = 22.6 μM, SI = 6.6) and ZIKV (EC_50_ = 27.9 μM, SI = 9.8). Compounds **13** significantly reduced viral production in HEK293T/17 (DENV) and A549 (ZIKV) cells, and proteomics revealed down-regulation of HSPA1A and up-regulation of PGK1 as key mechanistic differences from the parent andrographolide. Proteomics changes (e.g., HSPA1A, PGK1) are associative and do not prove causality without orthogonal validation.

Li et al. [62] designed compounds **14** (19-acetylated 14α-(5,7-dichloro-8-quinolyloxy)andrographolide, EC_50_ 4.5 μM, SI 19.7) and compounds **15** (14β-(8-quinolyloxy)-3,19-diol andrographolide, EC_50_ 1.3 μM, SI 17.5) as anti-ZIKV hybrids with low- to single-digit micromolar potency (Figure 4 presents representative antiviral andrographolide derivatives); the 5,7-dichloroquinoline and C-19 acetate of 14 lower cytotoxicity while the C-14 8-quinolyloxy and 3,19-diol pattern of **15** delivers maximal antiviral efficacy, jointly defining the andrographolide–quinoline platform for next-generation flavivirus inhibitors.

##### Anti-Chikungunya Virus Activity

Chikungunya is a mosquito-borne viral illness primarily spread by Aedes species mosquitoes. Initially identified in Tanzania in 1953, the causative agent—Chikungunya virus—belongs to the Alphavirus genus of the Togaviridae family. Typical clinical manifestations of the disease include an acute febrile phase accompanied by skin rash and severe joint pain, which often leads to significant immobility [63].

Against chikungunya virus, andrographolide derivatives exhibit antiviral activity by targeting viral enzymes and proteins that are essential for viral replication and assembly. The C-15 imine group and C-19 sulfonate group of these derivatives are crucial for their antiviral effects, as they can form covalent bonds with viral targets, thereby disrupting the virus life cycle.

Tran et al. [64] designed and synthesized compounds **16** (see Figure 4 for representative antiviral derivatives), EC_50_ = 0.14 μM, SI = 714, and compounds **17**, EC_50_ = 0.002 μM, SI = 12,715, as potent anti-CHIKV agents; both compounds effected ≥3.7-log viral reduction in HeLa CCL2 cells at 10–80 μM, maintained CC_50_ > 100 μM, and exhibited prophylactic as well as therapeutic efficacy against clinical CHIKV-122508 and CHIKV-6708 isolates, validating the oxindole-labdane hybrids as next-generation andrographolide-derived antivirals.

##### Anti-Enterovirus Virus Activity

Enterovirus 71 (EV71) was first isolated in California, USA, in 1969. This virus is closely related to poliovirus, with its specific receptors present on leukocytes, respiratory and gastrointestinal cells, and dendritic cells [65].

In the context of enteroviruses, andrographolide derivatives have been shown to inhibit the viral RdRp and 3CLpro, which are essential for viral RNA replication and polyprotein processing. The α, β-unsaturated lactone group of andrographolide derivatives is particularly effective in covalently binding to these viral targets, thereby inactivating them and reducing viral replication.

Kun Dai et al. [66]. synthesized the 14-aryloxy-8,17-epoxy andrographolide compound **18** (see Figure 4 for representative antiviral derivatives), which displayed potent anti-EV-A71 activity (IC_50_ = 0.95 μM, SI = 12.3). Compound **18** specifically inhibited post-entry viral RNA replication, significantly reduced VP0 and VP2 protein expression in RD cells, and exhibited broad-spectrum efficacy against CV-A16 (IC_50_ = 0.46 μM), CV-A6 (IC_50_ = 1.12 μM) and EV-D68 (IC_50_ = 1.59 μM); repeated passaging failed to yield resistant mutants, indicating host-factor targeting.

Jie Kai Tan et al. [67] prepared compound **19** (see Figure 4 for representative antiviral derivatives), bearing 14-quinolinoxy and 8,9-olefin modifications, that potently suppressed EV-A71 infection (IC_50_ = 2.06 μM, SI = 14.4). Compound **19** acted between 4–6 hpi to block viral RNA replication without affecting IRES-mediated translation, leading to marked loss of VP2 and 35-fold reduction in VP0; it also inhibited CV-A16, CV-A6, Echo7, CV-B5, CV-A24 and EV-D68, and no resistant mutants emerged after 21 passages, consistent with host-directed antiviral action.

#### 3.1.2. DNA Viruses

##### Anti-Hepatitis B Virus Activity

Since its identification in 1966, the hepatitis B virus (HBV) has been recognized as a major global health challenge, with over 350 million individuals affected. Chronic HBV infection is a primary etiological factor for serious liver conditions, including persistent hepatitis, liver cirrhosis, and hepatocellular carcinoma, contributing to approximately one million deaths each year [68].

Andrographolide derivatives have demonstrated the ability to inhibit the RdRp of HBV, which is crucial for the replication of the viral DNA. By targeting this enzyme, these compounds can effectively reduce the production of viral DNA, thereby limiting the spread of the virus within the host. Additionally, andrographolide derivatives may interfere with the virus–host protein interactions that are necessary for the virus to establish a persistent infection.

**Figure 5:**
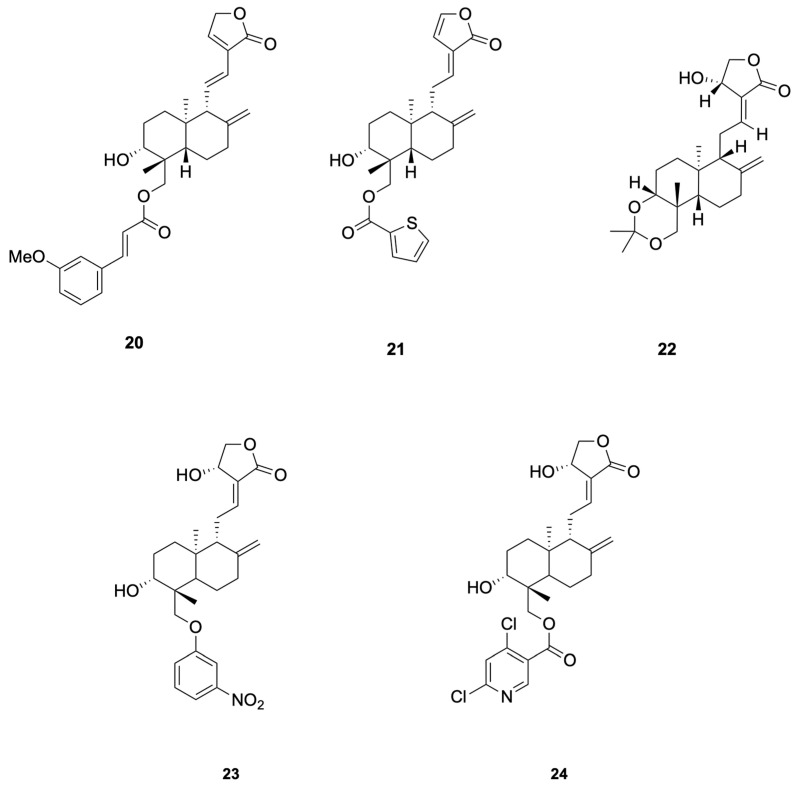
Compounds **20**–**24**.

Chen et al. [69] synthesized 48 dehydroandrographolide and andrographolide derivatives and identified compound **20** as the most potent inhibitor of HBV DNA replication (IC_50_ = 10.3 μM, SI > 165.1), while compound **21** simultaneously suppressed HBsAg, HBeAg secretion and DNA replication (SI = 20.3–104.9) (see Figure 5 for representative antiviral derivatives), corroborating the hypothesis that andrographolide scaffolds can interfere with HBV RdRp and viral–host protein interactions essential for persistent infection.

##### Anti-Herpes Simplex Virus Activity

Herpes simplex virus (HSV), a pathogen belonging to the Herpesviridae family, has been documented as early as ancient Greece. It commonly infects humans and can lead to diverse clinical outcomes, ranging from mild, self-limiting skin and mucous membrane lesions to severe, life-threatening conditions [70].

For herpes simplex viruses, andrographolide derivatives have been found to inhibit the viral RdRp and Mpro, similar to their action against other DNA viruses. These interactions prevent the virus from efficiently replicating its DNA genome and processing its polyproteins. The C-15 imine group and C-19 sulfonate group of andrographolide derivatives are particularly effective in covalently binding to viral targets, thereby inactivating them and reducing viral replication.

Priengprom et al. [71] synthesized Compound **22** and evaluated its anti-HSV activity (see Figure 5 for representative antiviral derivatives). Compound **22** suppressed HSV-1/HSV-2 cytopathic effects in Vero cells at non-cytotoxic concentrations (~20.5 μM) and lowered viral DNA and late proteins (e.g., ICP8/gD); time-of-addition/stage assays indicated a post-entry mechanism. Potency is in the low-to-single-digit tens micromolar range; standardized EC_50_/CC_50_/SI reporting and in vivo confirmation are still needed.

#### 3.1.3. Retroviruses

##### Anti-Human Immunodeficiency Virus Activity

The acquired immunodeficiency syndrome (AIDS) was initially documented in the summer of 1981. The causative agent, human immunodeficiency virus (HIV), was identified two years after the initial reports and subsequently confirmed in 1984 as the definitive etiology of AIDS [72].

Andrographolide derivatives have shown promise in inhibiting the RdRp and Mpro of HIV-1, which are essential for viral RNA replication and polyprotein processing. By targeting these enzymes, these compounds can effectively reduce the production of viral RNA and proteins, thereby limiting the spread of the virus within the host. Additionally, andrographolide derivatives may interfere with the virus–host protein interactions that are necessary for the virus to establish a persistent infection.

Uttekar et al. [73] synthesized a series of andrographolide derivatives and evaluated their anti-HIV activity. Among them, the 3-nitrobenzylidene derivative **23** exhibited higher in vitro anti-HIV activity (IC_50_ = 0.51 μM) compared to andrographolide (IC_50_ = 0.59 μM), while the 2,6-dichloro-nicotinoyl ester derivative **24** showed a higher Therapeutic Index (TI = 12,474) due to its lower cytotoxicity (see Figure 5 for representative antiviral derivatives). These derivatives inhibited gp120-mediated cell fusion, suggesting they interfere with early events in HIV entry by interacting with the V3 loop of gp120. Molecular docking studies revealed that these compounds bind to the V3 loop region of HIV-1 envelope protein, gp120, which is crucial for viral entry.

### 3.2. Antibacterial Activity

#### 3.2.1. Against Gram-Positive Bacteria

##### *Staphylococcus aureus* (MRSA)

*Staphylococcus aureus* is a pathogenic bacterium capable of inducing a wide spectrum of diseases, which may arise from either pyogenic mechanisms or toxin-mediated pathways. It is also a common causative agent in serious systemic and localized infections, including septicemia, infective endocarditis, pneumonia, as well as infections affecting the eyes and the central nervous system [74].

Andrographolide derivatives exhibit antibacterial activity against MRSA by increasing the production of reactive oxygen species (ROS) within the bacterial cells. This increase in ROS can lead to oxidative stress and damage to bacterial cellular components, ultimately resulting in cell death. Additionally, the membrane potential of MRSA cells is disrupted by andrographolide derivatives, which further contributes to their antibacterial effects. The C-14 ester chain length of andrographolide derivatives plays a significant role in determining their antibacterial potency against MRSA, with optimal chain lengths enhancing the interaction with the bacterial cell membrane.

**Figure 6:**
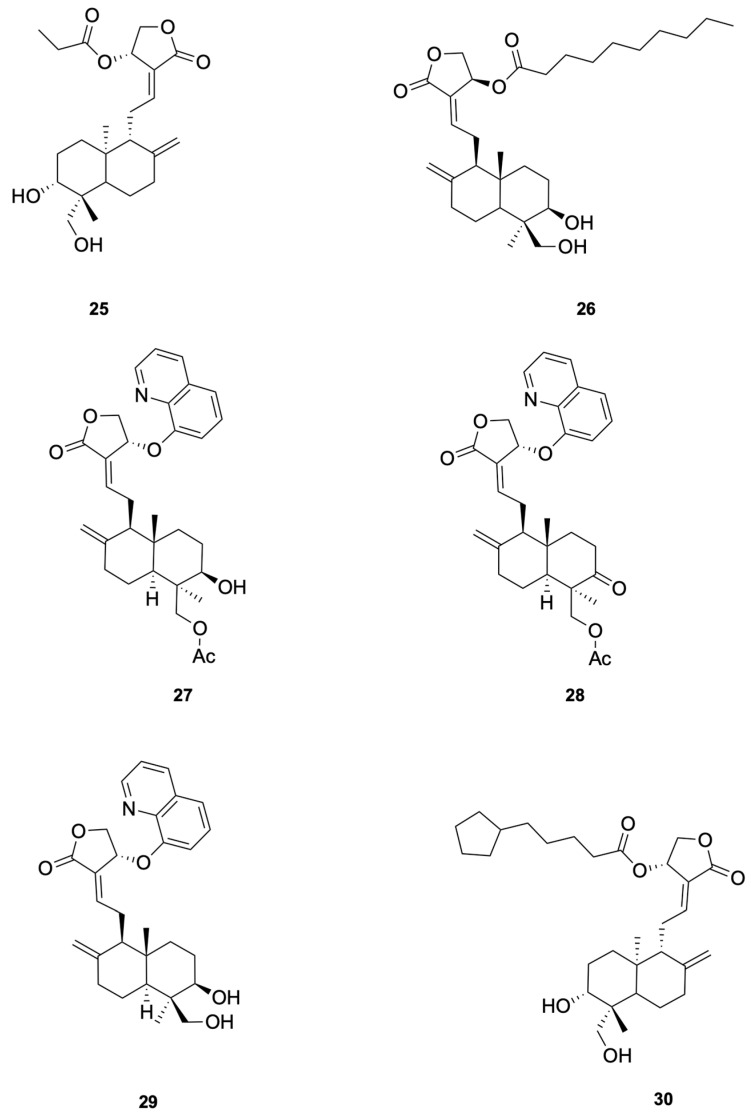
Compounds **25**–**30**.

Patil et al. [75] synthesized a series of andrographolide derivatives using Amano lipase AK (Pseudomonas fluorescens) as a biocatalyst to achieve stereoselective esterification, preferentially forming the propionyl ester of the S-isomer. Among the synthesized derivatives, compound **25** and compound **26** exhibited antimicrobial activity against *Staphylococcus aureus* with low minimal inhibitory concentration (MIC) values of 4 mg/mL and 16 mg/mL, respectively (see Figure 6 for representative antiviral derivatives). These derivatives also showed low hemolysis activity at their respective MICs and increased the permeability of the bacterial cell membrane, as demonstrated by FITC uptake and SEM imaging studies.

Hemolysis was evaluated at the MIC or multiples thereof; any hemolysis at MIC raises a toxicity concern for lead selection.

##### *Enterococcus* *faecalis*

*Enterococcus faecalis*, while naturally occurring as a Gram-positive symbiont in the intestinal flora of numerous species, has become a prominent cause of healthcare-associated infections. Its current status as a multidrug-resistant pathogen is a direct consequence of selective pressures from antibiotic use [76].

For *Enterococcus faecalis*, Feng et al. [77] synthesized a series of 14-aryloxy andrographolide derivatives. These derivatives were found to exhibit significant antibacterial activity against *E. faecalis*. The andrographolide skeleton was identified as a key structural feature for selectivity against *E. faecalis*, with the 14-aryloxy group, particularly the 14-(8-quinolinyloxy) group, emerging as a crucial pharmacophore. For example, compounds **27** and **28** demonstrated good selective inhibitory activity against *E. faecalis* (see Figure 6 for representative antiviral derivatives). These findings highlight the potential of andrographolide derivatives with specific structural modifications to serve as novel antibacterial agents against *E. faecalis*.

In our assays, this molecule was active mainly against Gram-positive bacteria and inactive/limited against the Gram-negative panel at the tested concentrations. However, related andrographolide derivatives bearing C-14 aryloxy substitution have shown Gram-negative activity (e.g., *E. coli*), with potency tracking with the C-14 ester/aryloxy chain length [77]. Thus, the current molecule appears narrow under our test conditions, whereas the andrographolide scaffold is substitution-dependent and tunable rather than universally narrow.

#### 3.2.2. Against Gram-Negative Bacteria

##### *Escherichia* *coli*

*Escherichia coli* (*E. coli*) commonly colonizes the human intestine as a nonpathogenic, facultative anaerobic commensal. Despite this, certain strains have acquired virulence factors that enable them to induce infections—including those affecting the gastrointestinal and urinary tracts, as well as the central nervous system—even in immunocompetent individuals [78].

To study the antibacterial activity against *Escherichia coli*, Feng et al. [77] synthesized a series of andrographolide derivatives. These derivatives were found to exhibit antibacterial activity against *E. coli* by increasing ROS production and disrupting membrane potential. These effects lead to oxidative stress and damage to bacterial cellular components, ultimately resulting in cell death. The C-14 ester chain length appears to correlate with antibacterial potency, although additional matched-pair studies are needed to confirm causality. For example, compound **29** (see Figure 6 for representative antiviral derivatives), which has an optimal C-14 ester chain length, demonstrated significant inhibitory activity against *E. coli*. These findings highlight the potential of andrographolide derivatives with specific structural modifications to serve as novel antibacterial agents against *E. coli*.

##### *Pseudomonas* *aeruginosa*

*Pseudomonas aeruginosa* is a bacterium that is widespread in the environment and can cause opportunistic infections in humans. Its diverse metabolic pathways and regulatory genes give it remarkable adaptability to different growth conditions. The bacterium’s nutritional flexibility, diverse virulence factors, and high antibiotic resistance make it extremely difficult to eradicate in infected hosts, especially in lung infections affecting patients with cystic fibrosis [79].

To study the anti-*Pseudomonas aeruginosa* activity, Wang et al. [80] designed and synthesized a series of andrographolide derivatives that inhibit bacterial quorum sensing (QS) and the production of virulence factors. These derivatives do not directly inhibit bacterial growth but interfere with QS signaling pathways, which are crucial for the regulation of virulence factors such as pyocyanin and protease. The study found that compound **30** (see Figure 6 for representative antiviral derivatives), a previously synthesized derivative, significantly inhibited the production of both pyocyanin and protease, suggesting that these compounds can disrupt QS-mediated virulence without directly killing the bacteria. The results highlight the potential of andrographolide derivatives as novel antibacterial agents that target QS pathways in *P. aeruginosa*.

### 3.3. Antifungal Activity

Derivatives caused a measurable increase in mitochondrial ROS (e.g., DCFH-DA assays) and modulated cell-wall integrity/MAPK signaling, consistent with antifungal activity. These derivatives primarily exert their antifungal effects through the induction of mitochondrial ROS production, which causes oxidative stress and damages fungal cell components. They also inhibit the cell wall integrity (CWI) pathway regulated by the MAPK signaling cascade, weakening the fungal cell wall. Additionally, the C-19 aryl ether substitution enhances their interaction with ergosterol in the fungal cell membrane, further disrupting membrane integrity. These mechanisms collectively contribute to the antifungal efficacy of andrographolide derivatives.

**Figure 7:**
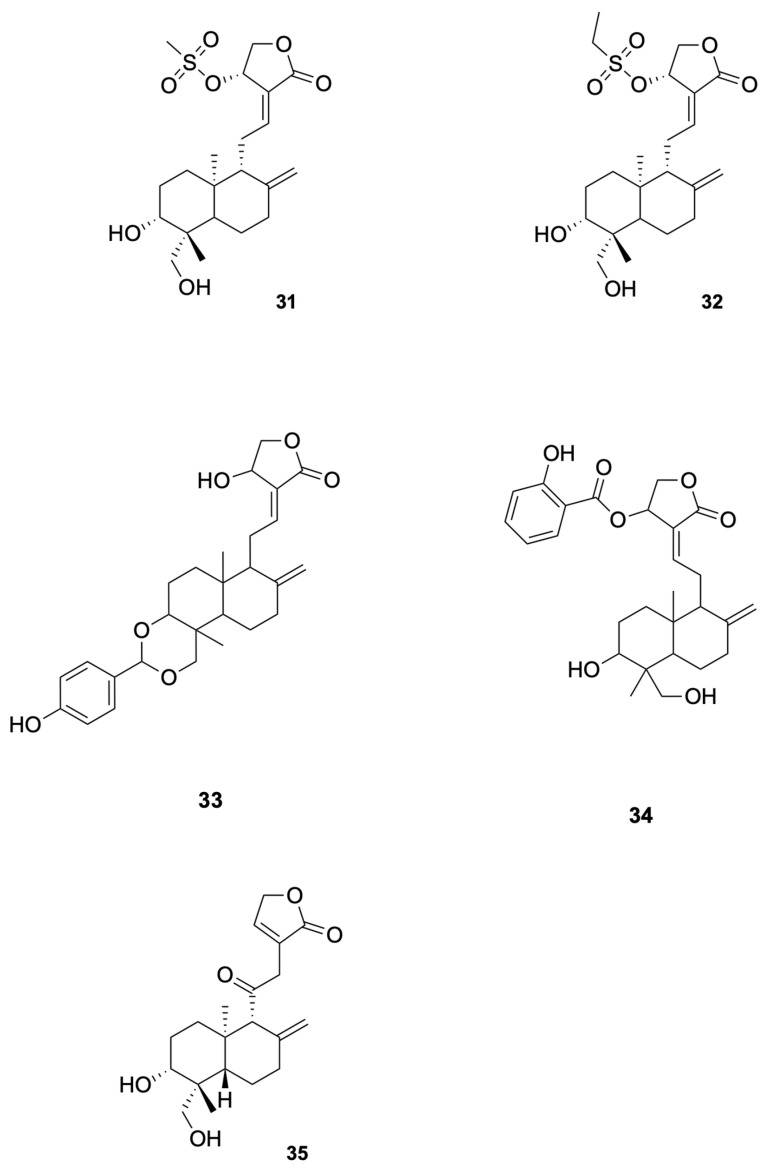
Compounds **31**–**35**.

Agarwal et al. [81] synthesized a series of C (14)-sulfonylester-type andrographolide derivatives and evaluated their antimicrobial activity against various bacterial and fungal strains. Among these derivatives, the methyl sulfonyl derivative compound **31** and ethyl sulfonyl derivative compound **32** exhibited significant inhibitory activity against fungal strains (see Figure 7 for representative antiviral derivatives), demonstrating the potential of structural modifications to improve antifungal efficacy. These results suggest that the introduction of sulfonylester groups at the C-14 position can enhance the antifungal activity of andrographolide derivatives.

### 3.4. Antiparasitic Activity

#### 3.4.1. *Plasmodium* spp. (Malaria)

Malaria, a life-threatening infectious disease with significant global impact on public health, is triggered by protozoan parasites from the Plasmodium genus. The transmission and propagation of this disease rely on an intricate life cycle that involves both mosquito vectors and vertebrate hosts [82]. Artemisinin-based combination therapies (ACTs) are the current frontline treatment for P. falciparum malaria. They combine a fast-acting artemisinin derivative with a partner drug [83]. Resistance has emerged especially in Southeast Asia, and cases are being increasingly reported in Africa. Resistance doesn’t always mean full failure of therapy, but often partial resistance manifested by delayed parasite clearance or persistence of parasites after certain time points in treatment [84].

Andrographolide derivatives have shown antiparasitic activity against *Plasmodium* spp., which are the causative agents of malaria. These derivatives can inhibit the mitochondrial electron transport chain of the parasite, thereby disrupt its energy metabolism and leading to parasite death. Additionally, andrographolide derivatives can inhibit the activity of topoisomerase II (TOPO II), which is essential for the replication of the parasite’s DNA. The C-15 long-chain alkyl substitution of andrographolide derivatives enhances their hydrophobicity, allowing them to more effectively insert into the parasite’s membrane and exert their antiparasitic effects [85,86,87].

Megantara et al. [88] reported that andrographolide derivatives, namely, compound **33** and compound **34**, exhibited high binding affinity with plasmepsin I, II, and IV (see Figure 7 for representative antiviral derivatives), which are key enzymes involved in the initial cleavage of hemoglobin in *Plasmodium* spp. The binding affinity values for compound 34 were −8.80 kcal/mol (plasmepsin I), −8.80 kcal/mol (plasmepsin II), and −8.30 kcal/mol (plasmepsin IV), indicating strong interactions with these enzymes. These results suggest that compound **33** and compound **34** have the potential to be developed into new antimalarial drugs. Docking/MD binding energies are hypothesis-generating and require confirmation by biochemical and cellular assays.

#### 3.4.2. *Leishmania* spp. (Leishmaniasis)

Leishmaniasis is a parasitic infection resulting from the transmission of intracellular *Leishmania parasites* through sand fly bites. The clinical presentation of the disease varies significantly based on the infecting species and the host’s immune status, spanning from self-limiting cutaneous lesions to systemic visceral involvement that can be life-threatening [89].

In the case of *Leishmania* spp., andrographolide derivatives exhibit antiparasitic activity by inhibiting the mitochondrial electron transport chain of the parasite. This inhibition disrupts the parasite’s energy metabolism, leading to its death. Additionally, andrographolide derivatives can inhibit topoisomerase II (TOPO II), which is essential for the replication of the parasite’s DNA. The C-15 long-chain alkyl substitution of andrographolide derivatives enhances their hydrophobicity, allowing them to more effectively insert into the parasite’s membrane and exert their antiparasitic effects. For example, studies have shown that andrographolide derivatives with C-15 alkyl chains significantly reduce the viability of Leishmania parasites by disrupting their mitochondrial function and inhibiting DNA replication. These mechanisms collectively contribute to the antiparasitic efficacy of andrographolide derivatives against *Leishmania* spp. [90,91].

Lala et al. [92] reported that the andrographolide derivative compound **35** showed significant antileishmanial activity (see Figure 7 for representative antiviral derivatives). In their study, the free form of the drug reduced the spleen parasite load by 39%, while the drug incorporated in liposomes, niosomes, and microspheres reduced the parasite load by 78%, 91%, and 59%, respectively. The highest efficacy was observed with niosomes, which reduced the parasite load by 91%. These results suggest that compound **35** has the potential to be developed into a new antileishmanial drug with enhanced efficacy and reduced toxicity.

The representative antiviral, antibacterial, antifungal, and antiparasitic data of andrographolide derivatives discussed in Section 3.1, Section 3.2, Section 3.3 and Section 3.4 are consolidated in Table S1 (see in Supplementary Materials).

## 4. Pharmacokinetics & Formulation Challenges

### 4.1. Oral Bioavailability: First-Pass Effect & β-Glucuronidation

Following oral administration, andrographolide exhibits low systemic exposure owing to extensive first-pass metabolism and limited solubility–permeability. [93,94,95]. In the intestine and liver, phase II conjugation (including β-glucuronidation) and oxidative biotransformation have been reported; in vitro interaction data indicate liabilities with UGT/CYP pathways that can further constrain exposure [95,96,97,98,99]. Microbiota-related transformations have also been observed in specific studies but may not be generalizable across settings [100,101]. Consequently, short half-life and relatively high clearance are frequently noted in preclinical models [93,94,95]. For oral dosing, the low observed toxicity may reflect limited systemic exposure due to poor PK (e.g., low solubility/permeability and first-pass metabolism); definitive safety assessment requires PK measurements (Cmax/AUC) at efficacious exposures.

### 4.2. Enabling Delivery to Enhance Exposure and Tissue Targeting

Liposomes. Phospholipid liposomes can encapsulate hydrophilic and lipophilic forms of andrographolide, stabilize the payload and prolong circulation, thereby increasing apparent exposure in vivo [102,103,104,105].

PLGA nanoparticles. Biodegradable PLGA-based systems (e.g., emulsion–solvent evaporation, nanoprecipitation) enable high loading, controlled release, and improved tissue distribution, increasing drug availability at infected sites in preclinical models [106,107,108,109,110].

β-Cyclodextrin inclusion. β-Cyclodextrin (β-CD) forms inclusion complexes with andrographolide that raise aqueous solubility and improve thermal stability [104].

Translation caveat. Andrographolide derivatives represent a versatile class of molecules with broad anti-infective potential and well-defined structure–activity relationships; biodistribution, hemocompatibility/hemolysis, complement activation, RES uptake, sterility/endotoxin, and dose proportionality should be characterized prior to translation [102,103,104,105,106,107,108,109,110,111].

### 4.3. Parenteral and Alternative Routes

Intravenous/intraperitoneal administration can circumvent first-pass metabolism and achieve exposures unattainable by oral dosing; formulation choice materially affects CL, V, and t½ [87,98]. Inhaled/intranasal delivery is conceptually attractive for respiratory infections (local lung exposure) but requires dedicated pulmonary safety and device/drug compatibility assessments before clinical application [95,96,97,98].

### 4.4. Exposure–Response and On-Target Engagement

To relate in vitro potency (EC_50_/IC_50_ in μM) to in vivo efficacy, studies should quantify free (unbound) plasma/tissue exposure, target engagement, and, where appropriate, biomarkers of pathway modulation. Minimal PK information includes IV/PO crossover, CL, V, t½, plasma protein binding (fu), metabolite identification (UGT/SULT/CYP), transporter liability, and tissue distribution in the relevant infection model [86,87,88,89,90,91]. Exposure-aware study design is essential to avoid over-interpreting biochemical/cellular potency.

### 4.5. Clinical and Regulatory Context

Andrographolide-containing preparations (e.g., andrographolide sulfonate/Xiyanping injection) are used clinically in some regions as herbal-derived products; this does not constitute regulatory approval of the purified parent compound as a stand-alone drug [33,40].

## 5. Conclusions

Andrographolide derivatives exhibit broad anti-infective potential with tractable structure–activity relationships (e.g., C-14/C-19 modifications and hybrid scaffolds). However, solubility and oral pharmacokinetics remain limited. Future efforts should focus on exposure-aware lead optimization, validation of delivery systems to achieve pharmacologically relevant concentrations, and bridging biochemical potency to in vivo efficacy.

## Figures and Tables

**Table 1:** Pathogen coverage and reporting standards used in this review.

Pathogen Class	Representative Microorganisms	Typical In-Vitro Systems	Primary Potency Metrics	Selectivity Metric	Mechanism/SAR Anchors (Examples)	Reference/Control Drug (≈Benchmark)
Viruses	SARS-CoV-2; influenza A/H1N1; DENV; ZIKV; CHIKV; EV-A71; HBV; HSV-1/2	Vero E6, A549, HEK293T/ACE2, Huh7, HepG2	EC_50_/IC_50_ (µM); CC_50_ (µM)	SI = CC_50_/EC_50_ (or IC_50_)	Mpro/RdRp/host-stress pathways; C-14/C-19 esters; hybrid labdane scaffolds	SARS-CoV-2: Remdesivir EC_50_ ≈ 0.77 µM; Chloroquine EC_50_ ≈ 2–5 µM [26,27]. Influenza A/H1N1: Oseltamivir (low-µM) [26]. DENV/ZIKV: Ribavirin ≈ 10–12 µM [26].
Bacteria	*Staphylococcus aureus* (MRSA); *Enterococcus faecalis*; *Escherichia coli*; *Pseudomonas aeruginosa*	Broth microdilution; time-kill; biofilm assays	MIC (µg·mL^−1^); CC_50_ (µM) when available	SI = CC_50_/EC_50_ (if available)	C-14 ester chain length vs. membrane interaction; energy metabolism pathways	MRSA: Ampicillin 1–8 µg/mL [28]. *E. coli*: Ciprofloxacin ≤ 0.25 µg/mL [27,28]. *P. aeruginosa*: Ceftazidime ≤ 1–4 µg/mL [29].
Fungi	*Candida albicans*; *Candida auris*; *Aspergillus fumigatus*	Yeast/mold susceptibility microplate; ROS (DCFH-DA) assays	MIC (µg·mL^−1^) or EC_50_/IC_50_ (µM); CC_50_ (µM)	SI (if reported)	Mitochondrial ROS↑; MAPK and cell wall stress pathways	*C. albicans*: Fluconazole ≤ 8 µg/mL [29]. *C. auris*: Fluconazole ≥ 32–256 µg/mL [30]. *A. fumigatus*: Amphotericin B ≈ 1 µg/mL [29].
Parasites	*Plasmodium falciparum*; *Leishmania donovani*	Asexual intraerythrocytic cycle; promastigote/amastigote assays	EC_50_/IC_50_ (µM); CC_50_ (µM)	SI = CC_50_/EC_50_	Computational docking/MD supported hypotheses; biochemical confirmation required	*P. falciparum*: Chloroquine 0.1–0.3 µM [31]. *L. donovani*: Amphotericin B < 1 µM [32].

## Data Availability

The original contributions presented in this study are included in the article and Supplementary Materials. Further inquiries can be directed to the corresponding author.

## Supplementary Materials

The following supporting information can be downloaded at: https://www.mdpi.com/article/10.3390/molecules30214273/s1, Table S1: Anti-infective activities of andrographolide derivatives (Compounds **4**–**35**).

## Abbreviations

The following abbreviations are used in this manuscript:

ARDS	Acute Respiratory Distress Syndrome
ACE2	Angiotensin-Converting Enzyme 2
Mpro	Main Protease
RdRp	RNA-dependent RNA Polymerase
HSPA1A	Heat Shock Protein A1A
PGK1	Phosphoglycerate Kinase 1
3CLpro	3C-like Protease
VP0	Viral Protein 0
VP2	Viral Protein 2
IRES	Internal Ribosome Entry Site
HBsAg	Hepatitis B Surface Antigen
HBeAg	Hepatitis B e Antigen
gp120	Glycoprotein 120
FITC	Fluorescein Isothiocyanate
SEM	Scanning Electron Microscopy
QS	Quorum Sensing
CWI	Cell Wall Integrity
TOPO II	Topoisomerase II
UGT	UDP-Glucuronosyltransferase
PLGA	Poly(Lactic-co-Glycolic Acid)
PEG	Polyethylene Glycol
DTA	Differential Thermal Analysis
TGA	Thermogravimetric Analysis
