# Supplementary material for: Andrographolide and Its Derivatives: A Comprehensive Review of Anti-Infective Properties and Clinical Potential
Source: Molecules. 2025 Nov 3;30(21):4273. doi: 10.3390/molecules30214273 (PMC12610367; doi:10.3390/molecules30214273)
Supplement: Supplementary file 1 [file molecules-30-04273-s001.zip › molecules-3906862-supplementary.pdf]

molecules

Review

Andrographolide and Its Derivatives: A Comprehensive Review of Anti-Infective Properties and Clinical Potential

Zimo Ren [1], Zihan Chen [1], Yuhan Xie [1] and Paolo Coghi [1,2,*]

[1] School of Pharmacy, Macau University of Science and Technology, Macau 999078, China; 2230028575@student.must.edu.mo (Z.R.); zhchen@must.edu.mo (Z.C.); 2240026307@student.must.edu.mo (Y.X.)
[2] State Key Laboratory of Mechanism and Quality of Traditional Chinese Medicine, Macau University of Science and Technology, 999078 Macao, China
* Correspondence: coghips@must.edu.mo; Tel.: +86-853-88972853

Academic Editor: Alexandru Mihai Grumezescu

Received: 16 September 2025
Revised: 28 October 2025
Accepted: 29 October 2025
Published: 3 November 2025

Citation: Ren, Z.; Chen, Z.; Xie, Y.; Coghi, P. Andrographolide and Its Derivatives: A Comprehensive Review of Anti-Infective Properties and Clinical Potential. *Molecules* **2025**, *30*, 4273. https://doi.org/10.3390/molecules30214273

Copyright: © 2025 by the author. Licensee MDPI, Basel, Switzerland. This article is an open access article distributed under the terms and conditions of the Creative Commons Attribution (CC BY) license (https://creativecommons.org/licenses/by/4.0/).

Table S1 Anti-infective activities of andrographolide derivatives compounds **4–35** (reference 46-85 are included in manuscript)

Compound (No.)	Pathogen / Target	Potency (EC_{50}/IC_{50}/MIC)	Selectivity Index (SI)	Ref. No.
**4**	Virus — SARS-CoV-2 (cell-based)	Cell antiviral activity shown (plaque reduction); EC_{50} not numerically reported	Not reported (no CC_{50}/TC_{50} available)	[46]
**5**	Virus — SARS-CoV-2 (cell-based)	Cell antiviral activity shown (plaque reduction); EC_{50} not numerically reported	Not reported (no CC_{50}/TC_{50} available)	[46]
**6**	Virus — SARS-CoV-2 (Mpro, enzyme)	IC_{50} = 15.05 ± 1.58 μM (Mpro, enzyme)	Not applicable (enzyme assay; no CC_{50}/TC_{50})	[47]
**7**	Virus — SARS-CoV-2 (Mpro, enzyme)	IC_{50} = 2.79 ± 0.22 μM (Mpro, enzyme; Andro-NBD)	Not applicable (enzyme assay; no CC_{50}/TC_{50})	[47]
**8**	Virus — SARS-CoV-2 (Mpro, enzyme)	>50% inhibition at 10 μM (best hits in series)	Not applicable (enzyme assay; no CC_{50}/TC_{50})	[48]
**9**	Virus — SARS-CoV-2 (Mpro, enzyme)	>50% inhibition at 10 μM (best hits in series)	Not applicable (enzyme assay; no CC_{50}/TC_{50})	[48]
**10**	Virus — SARS-CoV-2 (Mpro, enzyme)	>70% inhibition at 100 μM (Mpro, enzyme; C-12 dithiocarbamate analogue)	Not applicable (enzyme assay; no CC_{50}/TC_{50})	[45]
**11**	Virus — SARS-CoV-2 (Mpro, enzyme)	>70% inhibition at 100 μM (Mpro, enzyme; C-12 dithiocarbamate analogue)	Not applicable (enzyme assay; no CC_{50}/TC_{50})	[45]
**12**	Unspecified (see cited reference)	Not reported in the cited source	Not reported or not computable	[50]
**13**	Virus — Dengue-2 (cell-based)	EC_{50} = 21.3–22.7 μM (HepG2/HeLa)	Not reported (no CC_{50}/TC_{50} available)	[54]
**14**	Virus — Zika (cell-based)	EC_{50} = 1.3 μM; CC_{50} = 20.9–22.7 μM	SI > 16	[55]
**15**	Virus — Zika (cell-based)	EC_{50} = 27.9 ± 1.7 μM (ZAD-1); 31.8 ± 3.5 μM (andrographolide)	Not reported (no CC_{50}/TC_{50} available)	[55]
**16**	Virus — Chikungunya virus (CHIKV-122508; HeLa CCL2, cell-based)	EC_{50} = 0.02 μM; CC_{50} = 15.2 μM (post-treatment, 24 h, plaque assay)	758	[57]

**17**	Virus — Chikungunya virus (CHIKV-122508; HeLa CCL2, cell-based)	EC_{50} = 0.03 μM; CC_{50} = 13.8 μM (post-treatment, 24 h, plaque assay)	461	[57]
**18**	Virus — Enterovirus A71 (cell-based)	IC_{50} = 4.44 μM; CC_{50} = 29.91 μM (ZAF-46)	≈ 6.7	[59]
**19**	Virus — Enterovirus A71 (cell-based)	IC_{50} = 2.06 μM; CC_{50} = 29.57 μM (ZAF-47)	≈ 14.35	[60]
**20**	Virus — HBV (HepG2.2.15)	IC_{50} = 54.07 μM; SI = 3.7 (HBV DNA replication)	3.7	[62]
**21**	Virus — HBV (HepG2.2.15)	IC_{50} = 22.58 μM; SI = 8.7 (HBV DNA replication)	8.7	[62]
**22**	Virus — HSV-1 (cell-based)	Marked suppression of ICP8 expression at ~22.5 μM; standard EC_{50} not reported	Not reported (no CC_{50}/TC_{50} available)	[64]
**23**	Virus — HIV-1 (cell-based)	EC_{50} = 49.0 μg/mL (andrographolide); 56.8 μg/mL (14-deoxy-11,12-didehydro-AGL)	Not reported (no CC_{50}/TC_{50} in source)	[66]
**24**	Virus — HIV-1 (cell-based)	EC_{50} = 49.0 μg/mL (andrographolide); 56.8 μg/mL (14-deoxy-11,12-didehydro-AGL)	Not reported (no CC_{50}/TC_{50} in source)	[66]
**25**	Bacteria — *Pseudomonas aeruginosa* (quorum-sensing)	Quorum-sensing phenotype inhibition; numeric % provided in Remarks (not MIC)	Not applicable (virulence modulation; no CC_{50}/TC_{50})	[68]
**26**	Bacteria — *Pseudomonas aeruginosa* (quorum-sensing)	Quorum-sensing phenotype inhibition; numeric % provided in Remarks (not MIC)	Not applicable (virulence modulation; no CC_{50}/TC_{50})	[68]
**27**	Bacteria — *Pseudomonas aeruginosa* (quorum-sensing)	Quorum-sensing phenotype inhibition; numeric % provided in Remarks	Not applicable (virulence modulation; no CC_{50}/TC_{50})	[70]
**28**	Bacteria — *Pseudomonas aeruginosa* (quorum-sensing)	Quorum-sensing phenotype inhibition; numeric % provided in Remarks	Not applicable (virulence modulation; no CC_{50}/TC_{50})	[70]
**29**	Bacteria — *Pseudomonas aeruginosa* (quorum-sensing)	Quorum-sensing phenotype inhibition; numeric % provided in Remarks	Not applicable (virulence modulation; no CC_{50}/TC_{50})	[70]
**30**	Bacteria — *Pseudomonas aeruginosa* (quorum-sensing)	Quorum-sensing phenotype inhibition; numeric % provided in Remarks	Not applicable (virulence modulation; no CC_{50}/TC_{50})	[73]
**31**	Bacteria — *E. coli*; K. pneumoniae; S. aureus; B. subtilis (MIC; liquid dilution)	MIC (μg/mL): 4a = 2.5/5/5/2.5; 4b = 5/5/2.5/5; 4c = 30/25/25/35; 4d = 20/15/30/20; 4e = 15/25/30/20; 4f = 30/30/35/30; 4g = 25/15/20/5; AGL = 10/15/15/5; Ampicillin = 5/5/10/5	Not computable (no CC_{50}/TC_{50} reported)	[74]

**32**	Bacteria — *E. coli*; K. pneumoniae; S. aureus; B. subtilis (MIC; liquid dilution)	MIC (μg/mL): 4a = 2.5/5/5/2.5; 4b = 5/5/2.5/5; 4c = 30/25/25/35; 4d = 20/15/30/20; 4e = 15/25/30/20; 4f = 30/30/35/30; 4g = 25/15/20/5; AGL = 10/15/15/5; Ampicillin = 5/5/10/5	Not computable (no CC_{50}/TC_{50} reported)	[74]
**33**	Parasite — *Plasmodium falciparum* plasmepsin I/II/IV (in silico docking)	Binding affinity ($kcal·mol^{-1}$) / calc Ki (μM): AGL −7.7/−7.7/−6.3; AND10 −8.5/−8.5/−7.6 (Ki ≈ 0.59/0.59/2.70); AND15 −8.8/−8.8/−8.3 (Ki ≈ 0.36/0.36/0.83)	Not applicable (computational docking)	[81]
**34**	Parasite — *Plasmodium falciparum* plasmepsin I/II/IV (in silico docking)	Binding affinity ($kcal·mol^{-1}$) / calc Ki (μM): AGL −7.7/−7.7/−6.3; AND10 −8.5/−8.5/−7.6 (Ki ≈ 0.59/0.59/2.70); AND15 −8.8/−8.8/−8.3 (Ki ≈ 0.36/0.36/0.83)	Not applicable (computational docking)	[81]
**35**	Parasite — Leishmania donovani (in vivo, hamster)	Spleen parasite-load reduction at 2 $mg·kg^{-1}$ s.c., every 3 days ×6: free drug 39%; liposomal 78%; niosomal 91%; microsphere 59%	Not computable (no CC_{50}/TC_{50} reported)	[85]
